# Supplementary material for: Can Teledentistry Replace Conventional Clinical Follow-Up Care for Minor Dental Surgery? A Prospective Randomized Clinical Trial
Source: Int J Environ Res Public Health. 2022 Mar 15;19(6):3444. doi: 10.3390/ijerph19063444 (PMC8953526; doi:10.3390/ijerph19063444)
Supplement: Supplementary file 1 [file ijerph-19-03444-s001.zip › ijerph-1630989-supplementary.pdf]

Table S1. Descriptives of surgeries performed during the study.

Dentoalveolar Surgery	Telephone follow-up		Conventional follow-up		Total		*p*
Tooth extraction	**n**	**%**	**n**	**%**	**n**	**%**	
	21	63.6	20	57.1	41	60.3	0.587
Osteotomy Wisdom tooth	9	27.3	8	22.9	17	25	0.677
Osteotomy	3	9.1	7	20	10	14.7	0.208
Suture	22	66.7	26	74.3	48	70.6	0.494
Teeth	**Mean**	**SD**	**Mean**	**SD**	**Mean**	**SD**	
	2	2.3	1.8	1.5	1.9	1.9	0.910

Table S2. Results of the questionnaire for both groups.

Question	Telephone follow-up		Conventional follow-up		Total		*p*
Feeling sick [yes/no]	**n**	**%**	**n**	**%**	**n**	**%**	
	2/28	6.7/93.3	0/30	0/100	2/58	3.3/96.7	0.154
Pain medication [yes/no]	5/25	16.7/83.3	5/25	16.7/83.3	10/5	16.7/83.3	1.00
Daily activity [yes/no]	27/3	90/10	27/3	90/10	54/6	90/10	1.00
Concerns [yes/no]	4/26	13.3/86.7	1/29	3.3/96.7	5/55	8.3/91.7	0.165
Soft food [yes/no]	11/19	36.7/63.3	15/15	50/50	26/3	43.3/56.7	0.301
Bleeding [yes/no]	0/30	0/100	1/29	3.3/96.7	1/59	1.7/98.3	0.317
Swelling/Fever [yes/no]	1/29	3.3/96.7	2/28	6.7/93.3	3/57	5/95	0.557
Problems swallowing/breathing [yes/no]	0/30	0/100	0/30	0/100	0/60	0/100	1.00
Satisfied [yes/no]	30/0	100/0	30/0	100/0	60/0	100/0	1.00
Remaining questions [yes/no]	10/20	33.3/66.7	5/25	16.7/83.3	15/4	25/75	0.139
Wish face-to-face follow-up [yes/no]	5/25	16.7/83.3	12/18	40/60	17/4	28.3/71.7	0.047
Long travel time [yes/no]	–	–	18/12	60/40			
Face-to-face follow-up needed [yes/no]	2/28	6.7/93.3	3/27	10/90	5/55	8.3/92,7	0.643
Requirement to treat complication	2/28	6.7/93.3	3/27	10/90	5/55	8.3/92,7	0.634
Waiting time [min]	**Mean**	**SD**	**Mean**	**SD**	**Mean**	**SD**	
	–	–	12.8	11.5			
Duration of appointment [min]	3.9	2.1	4.7	3.9	4.3	3.1	0.178
